# Structure of cytotoxic associated antigen A protein of *Helicobacter pylori* from Bali and Lombok isolates of Indonesia

**DOI:** 10.14202/vetworld.2020.1319-1326

**Published:** 2020-07-13

**Authors:** Hamong Suharsono, Dewa Nyoman Wibawa, Zainul Muttaqin, Kadek Karang Agustina

**Affiliations:** 1 Laboratory of Biochemistry, Faculty of Veterinary Medicine, Udayana University, Denpasar, Indonesia; 2 Internal Medicine Laboratory, Faculty of Medicine Udayana University, Denpasar, Indonesia; 3 Biomedical Research Unit, West Nusa Tenggara General Hospital, Lombok, Indonesia; 4 Department of Veterinary Public Health, Faculty of Veterinary Medicine Udayana University, Denpasar, Indonesia

**Keywords:** Bali, cytotoxic associated antigen A, *Helicobacter pylori*, Lombok, structure, virulence

## Abstract

**Background and Aim:**

*Helicobacter* *pylori* is a well-known zoonotic agent with worldwide distribution. In Indonesia, only one report regarding the variation within the cytotoxic associated antigen A (CagA) protein of *H. pylori* has been described in the literature, which was conducted in Manado, South Sulawesi. There remains no report concerning the structure of this protein, particularly for the Bali and Lombok isolates. The objective of this study was to investigate the diversity o*f H. pylor*i CagA amino acid sequences of Bali and Lombok isolates, to predict their molecular structures and conduct toxicity examination of CagA on gastric cells.

**Materials and Methods:**

A total of 36 samples were used in equal proportions for each pathologic condition. DNA extraction was performed to subculture *H. pylori* Bali isolates. The amplification of the CagA 3′ variable region was carried out using the primers P1 (5′-GATAACAGGCAAGCTTTTTGAGG-3′) and P2 (5′-CTGCAAAAGATTGTTTGGCAG-3′). The W2, W9, and W35 fragments were selected as a representation of *H. pylori* Bali isolates, which were modeled through the threading modeling approach using I-TASSER.

**Results:**

According to the 12 CagA sequences obtained and phylogenetic analyses, the *H. pylori* strain originating from Bali can be grouped within the East Asian genotypes and is identical to the Lombok strain. In addition, the Bali isolates are phylogenetically more closely related to Southeast Asian strains, particularly the Filipino strain. The relationship between degree of inflammation induced and CagA-positive infection was not statistically significant.

**Conclusion:**

The structure of the *H. pylori* Bali isolate is identical to that of Lombok isolate, which belongs to the same group of East Asian genotypes, and bacterial virulence is not related to structure.

## Introduction

*Helicobacter pylori* was first reported to cause infection within human gastric mucosa in 1983 [1]. This was followed by the discovery of other strains of *H. pylori* in the gastric mucosa of various animals, including: *Helicobacter muridarum* in mice, *Helicobacter felis* in cats, *Helicobacter nemestrinae* in squirrels, and *Helicobacter canis* in dogs [2,3]. It is estimated that more than half of the world’s population have been infected by this microaerophilic organism [4], with a variety of prevalence among industrialized and developing countries. In Indonesia, the prevalence of *H. pylori* infection is approximately 53.8% [5]. A similar result was reported in Mataram, Lombok where ~ 53.4% of blood donors tested positive for anti-*H. pylori* antibodies [6]. This data were supported by Soemohardjo *et al*. [7], in which *H. pylori* isolates were detected within gastric tissue of ~ 42.9% of patients through polymerase chain reaction (PCR) method. Clinical manifestation of *H. pylori* colonization within the stomach allegedly exhibits a strong correlation with genotype or genetic variation of the pathogenic bacteria [8]. Researchers have identified several DNA sequences associated with gastric cancer, for example: The DNA motif (AATAAGATA) on the 5’ region of cytotoxic associated antigen A (CagA), and the repeated region on the 3’ end of CagA within the East Asian subtype. In addition, it was found that the phylogenetic origins of bacterial strains also affect the carcinogenic potential of *H. pylori* infection [9].

The most important virulence factors related to *H. pylori* infection are expression of the *CagA* and vacuolating cytotoxic antigen (*VacA*) genes, as well as the urease enzyme [10]. *H. pylori* is known to be one of the most genetically diverse microorganisms [11] and was initially divided into two strains: The virulent strain one that produces CagA and VacA proteins, and the non-virulent strain two which does not produce these two toxin proteins [12,13]. Following the identification of a variant allele within CagA at the C-terminal of the protein, *H. pylori* was categorized into East Asian and Western strains. The East Asian strain is predominantly found in Japan, China, and South Korea, whereas the Western strain is common to the United States, Europe, and Australia [4]. At the protein level, Covacci *et al*. [14] reported that a protein with molecular weight of 128 kDa is associated with the incidence of cytotoxicity and peptic ulcer. Moreover, analysis of a variant of the CagA 5’ region resulted in significant correlation with the origin and type of gastric disease that occurred [15].

The CagA protein influences cell activity through alteration of host cell signaling pathways, particularly concerning the presence of the EPIYA amino acid sequence located in the C-terminal of CagA protein – consisting of Glu-Pro-Ile-Tyr-Ala – with the tyrosine residue serving as the phosphorylation site. There exist several reduplications of different motifs within the *CagA* gene, including EPIYA-A, -B, -C, and -D. EPIYA-A and EPIYA-B are always present in CagA-positive cases. However, the numbers and arrangement of the two other segments vary according to bacterial strain and its pathogenicity. Segments categorized as type EPIYA-A, -B, and -D are present in the East Asian strain found in Central Asia, East Asia, and North America and have been reported to be highly associated with increased incidence of gastric cancer. Meanwhile, the western strain consists of EPIYA-A, -B, and -C motifs and can be found in Western and Middle Eastern countries, where motif C may have been duplicated several times and differs from one geographical region to another [16].

Studies conducted in several countries such as Turkey, the United States, and Japan showed that variation in CagA-EPIYA is associated with the occurrence of gastric pathology. In Indonesia, there has been only one report regarding variation of the *H. pylori* CagA protein, which was conducted in Manado, South Sulawesi; the incidence, however, was exceptionally low [17,18] within Lombok isolates. Toxicity of CagA is determined by the molecular structure of the C-terminal of the protein. The stronger it binds to the MAP kinase SHP2 [19], the more toxic effects may be elicited.

The objective of this study was to investigate the diversity of *H. pylori* CagA amino acid sequences of Bali and Lombok isolates, to predict their molecular structures and conduct toxicity examination of CagA on gastric cells.

## Materials and Methods

### Ethical approval

This research was approved by the Animal Ethics Committees of The Faculty of Veterinary Medicine at Udayana University, Bali (Ref. No. 1167/UN14.2.9/PD/2019).

### Samples

The study was conducted from April to May 2019 at the Sanglah Hospital and involved a total of 36 subjects; the samples were collected from patients who met the inclusion criteria by obtaining tissue samples from gastric body and antrum through biopsy. The study subjects were comprised of 22 (61.11%) male and 14 (38.89%) female subjects between the ages of 25 and 82 years. Clinical diagnosis of the study participants included gastritis, gastric ulcer, and gastric neoplasms.

The experimental study was conducted using a random group approach with CagA protein structure as the dependent variable and gastric mucosal injury as the independent variable. Gastric pathology was categorized into three types of disease, including: Gastritis, gastric/duodenal ulcer, and gastric carcinoma. The samples were divided into equal proportions for each pathologic condition.

Subcultures of *H. pylori* Bali isolates were obtained from cultures of gastric biopsy specimens of gastric body, antrum, and pylorus of patients presenting with dyspepsia symptoms to the Internal Medicine Outpatient Clinic of Sanglah Central Hospital. *H. pylori* isolates (n=36) were stored in Trypticase Soy Broth medium with 10% glycerol at −80°C at Biomedical Research Unit Laboratory, West Nusa Tenggara Regional Hospital. *H. pylori* was subsequently subcultured using Trypticase Soy Agar medium supplemented with 10% sheep’s blood, 2 mL/500 mL Dent medium and 10 mL/500 mL Vitox. The culture was then incubated in a CO_2_ incubator to produce microaerophilic environment with 5% O_2_, 10% CO_2_, and 85% N_2_ for 48 h at 37°C temperature.

### DNA extraction

DNA extractions of bacterial cells were prepared for purification using DNAzol reagent (Invitrogen^®^). Approximately 1 × 10^9^ cells in 1 mL were centrifuged at 10,000 rpm for 5 min, and the supernatant was then removed. Five hundred microliters of DNAzol reagent was inverted 10 times and then vortexed at 200 rpm for 2 min. This sample was centrifuged at 10,000 rpm for 10 min; and the supernatant was transferred to a new sterile tube. Five hundred microliters of absolute ethanol solution were added to the supernatant, and the mixture was subsequently incubated at room temperature for ~ 3 min, followed by mixing of the inversion portion and centrifugation at 4000 rpm for 5 min. The supernatant was carefully removed, and the remaining pellet was washed twice with 80% ethanol. The pellet was resuspended with either 50 μL dd H_2_O or 8 mM NaOH solution.

### Amplification and sequencing of *CagA* DNA

Genomic DNA extraction was performed using the DNAZol Kit(Invitrogen, Thermo Fisher Scientific, California, US), according to the manufacturer’s instructions. The amplification of the *CagA* 3′ variable region was carried out using the primers P1 (5′-GA TAACAGGCAAGCTTTTTGAGG-3′) and P2 (5′CTGCAAAAGATTGTTTGGCAG-3′) (18). The amplification steps were conducted under the following conditions: 94°C for 1 min; 34 cycles of 94°C for 1 min, and 55°C for 1 min, followed by 72°C for 1 min; with a final extension at 72°C for 5 min. The solutions were then stored at 4°C. PCR products were separated by 2% agarose gel electrophoresis and examined under ultraviolet illumination, then sequenced. The data presented as sequences either resulting from the sequencing process or downloaded from GenBank were analyzed using BioEdit and MEGAv7 software [20]. The phylogenetic tree was constructed using a maximum likelihood algorithm on 36 *CagA* sequences of Bali origin and eight isolate sequences obtained from neighboring countries downloaded from the GenBank.

### *In silico* analysis

Three *CagA* fragments were chosen as representation of *H. pylori* Bali isolates – namely, W2, W9, and W35 fragments – which were modeled through threading modeling approach using I-TASSER, which is an online platform that can estimate the three-dimensional structure of a protein as well as predict its function. The three-dimensional model of the targeted protein generated by the I-TASSER server results from a combination of the predictions obtained from the multiple-threading alignment process. The prediction result of the model is chosen based on the confidence score (c-score) [21]. Subsequently, the model was reconstructed using ModRefiner to obtain a reliable model, the quality of which was then assessed based on a Ramachandran Plot as analyzed using RAMPAGE. Meanwhile, the SHP2 (PDBID: 2SHP) model was obtained using experimental X-ray diffraction with 2A resolution which was downloaded from the Protein Data Bank. Then SHP2 was modeled as a receptor with the *Lom* fragment serving as ligand, both of which subsequently underwent docking using PATCHDOCK [22]. This docking process was conducted rigidly and was followed by the refinement process of flexible docking using FIREDOCK. The flexibly conducted docking process followed actual conditions or used induced fit interaction principles. Docking was performed blind as the binding site of the ligand on the receptor remained unknown. The resulting model was chosen based on that with the most negative binding energy, as the more negative the binding energy, the higher the affinity of the ligand toward its receptor.

### Statistical analysis

The data were analyzed using SPSS program for Windows version 24 (IBM Corp., NY, USA). Discrete variables were tested using a Chi-square test, whereas continuous variables were tested using the Mann–Whitney U- and t-tests [23]. A two-tailed p<0.05 was considered statistically significant [24]. Data for the CagA C-terminal structure patterning and SHP2 binding intensity were analyzed using non-parametric analysis.

## Results

### Baseline characteristics of study population

**Figure-1:**
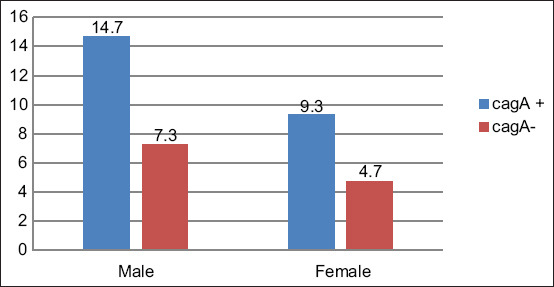
Comparison of cytotoxic associated antigen A testing results based on sex; p=0.003, with Fisher’s exact test = 0.003, and an alpha value of 0.05 and probability of 90.0%.

**Figure-2:**
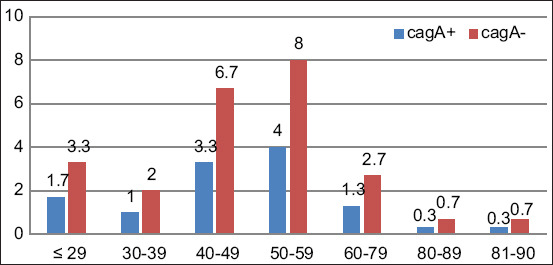
Comparison of cytotoxic associated antigen A testing results based on age group; p=0.136, with Fisher’s exact test = 0.142, and an alpha value of 0.05, and probability of 90.0%.

**Figure-3:**
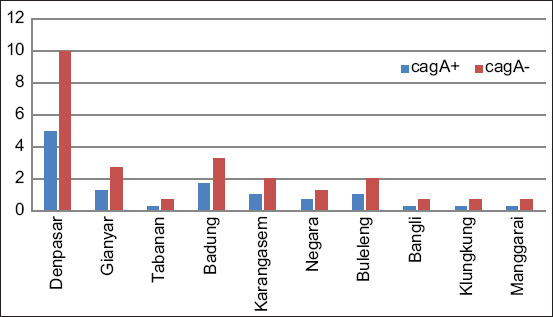
Comparison of cytotoxic associated antigen A testing results based on demographic background; p=0.937, with fisher exact test = 0.951 and alpha value of 0.05 and probability of 90.0%.

**Figure-4:**
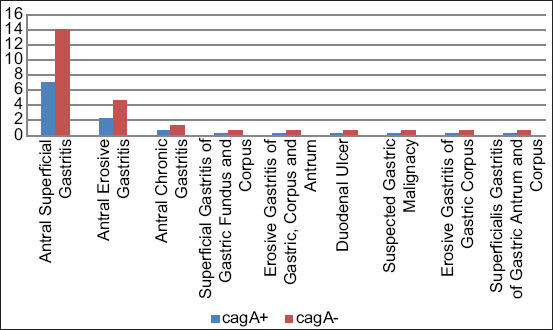
Comparison of cytotoxic associated antigen A testing results based on the results of endoscopic study; p=0.663, with Fisher’s exact test result = 0.672 and alpha value of 0.05 and probability of 90.0%.

**Figure-5:**
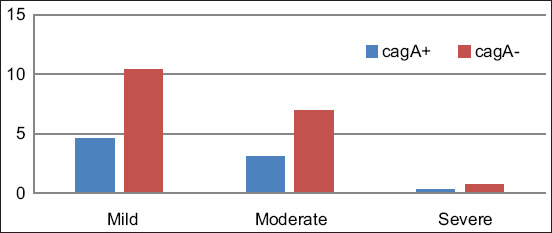
Comparison of cytotoxic associated antigen A testing results based on the degree of inflammation; p=0.769, with Fisher’s exact test result of 0.769 and alpha value of 0.05 and probability of 90.0%.

In this study, 12 (33.3%) participants tested positive for CagA protein. The results of the endoscopic studies conducted on study participants showed a variety of gastric pathology, including: Antral superficial gastritis in 21 (58.33%), antral erosive gastritis in 7 (19.44%), antral chronic gastritis in 2 (5.55%), superficial gastritis involving gastric fundus and corpus in 1 (2.78%), erosive gastritis involving gastric corpus and antrum in 1 (2.78%), duodenal ulcer in 1 (2.78%), suspected gastric malignancy in 1 (2.78%), erosive gastritis of the gastric corpus in 1 (2.78%), and superficial gastritis involving gastric antrum and corpus in 1 (2.78%). To simplify, the proportion of study participants exhibiting gastric pathology are as follows: Gastritis in 34 (94.44%), duodenal ulcer in 1 (2.78%), and gastric malignancy in 1 (2.78%) (Table-1 and Figures-1-5).

**Table-1:** Demographic characteristics of study participants.

Variable	n (%)
Sex	
Male	22 (61.11)
Female	14 (38.89)
Age group	
≤29	5 (13.89)
30-39	3 (8.33)
40-49	10 (27.78)
50-59	12 (33.33)
60-69	4 (11.11)
70-79	1 (2.78)
80-89	1 (2.78)
Demographic background	
Denpasar	15 (41.67)
Gianyar	4 (11.11)
Tabanan	1 (2.78)
Badung	5 (13.89)
Karangasem	3 (8.33)
Negara	1 (2.78)
Buleleng	3 (8.33)
Bangli	1 (2.78)
Klungkung	1 (2.78)
Manggarai	1 (2.78)
Endoscopic features	
Antral superficial gastritis	21 (58.33)
Antral erosive gastritis	7 (19.44)
Antral chronic gastritis	2 (5.55)
Superficial gastritis involving gastric fundus and corpus	1 (2.78)
Erosive gastritis involving gastric corpus and antrum	1 (2.78)
Duodenal ulcer	1 (2.78)
Suspected gastric malignancy	1 (2.78)
Erosive gastritis of gastric corpus	1 (2.78)
Superficial gastritis involving gastric corpus and antrum	1 (2.78)

### PCR of *CagA* genes

**Figure-6:**
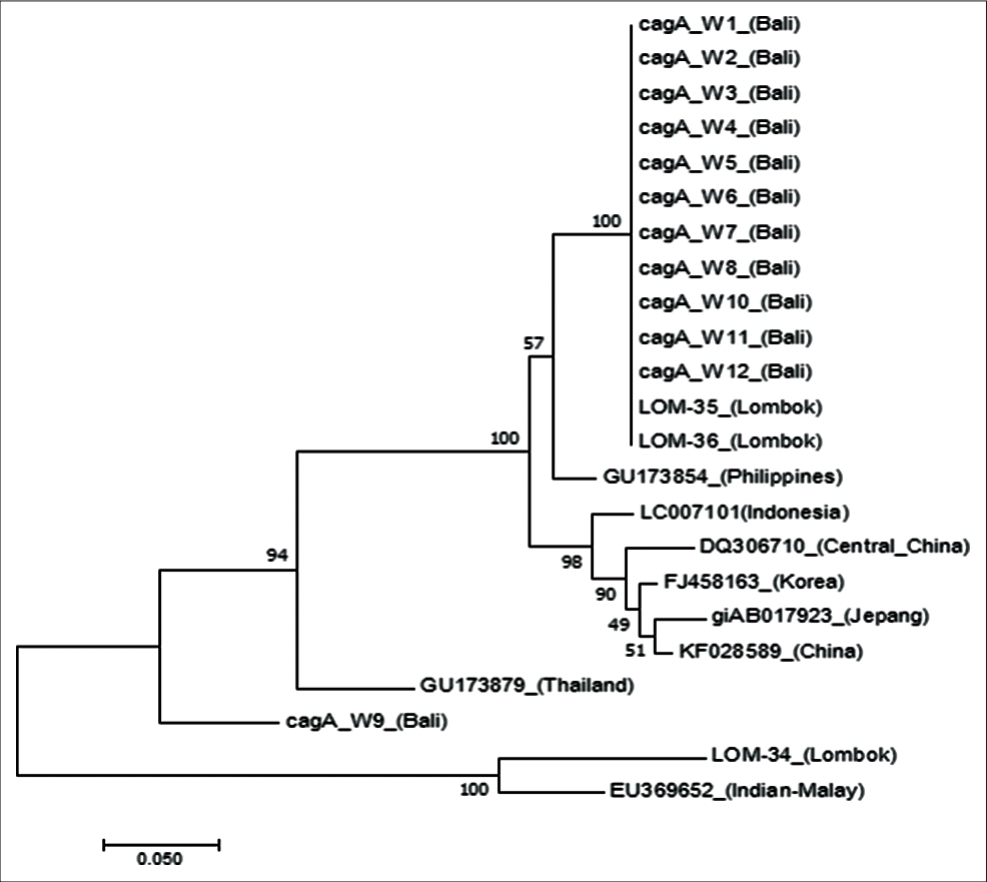
Phylogenetic tree built using maximum likelihood (ML) algorithm from cytotoxic associated antigen A genes of *Helicobacter pylori* Bali isolates (W1 to W12), *H. pylori* Lombok isolates (LOM34, LOM35, and LOM36), as well as *H. pylori* from several Asian countries. The numbers on the branches represent the percentage of bootstrap used.

From 20 biopsy tissues within paraffin blocks and 30 specimens obtained from gastric mucosa biopsy, 12 specimens tested positive for *CagA* using PCR which included several genes, namely: M1334.17, M1499.17, M1606.17, and M1858.17 (hereafter referred to as W1, W2, W3, and W12) (Figure-6).

## Discussion

The prevalence of positive CagA results was 33.33% of the 36 study participants, with the prevalence in men (61.11%) being significantly higher than in women (38.89%) (Table-1). These results are in accordance with a previous study conducted by Kaore *et al*. [25], which also showed a higher prevalence in men compared to women. The results indicated a very significant association between sex and prevalence of CagA-positive *H. pylori* infection with p=0.003. These data, therefore, indicate that CagA-positive infection is influenced by gender differences (Figure-1).

The mean age of the infected patients was in the range of 50-59 years. There was no significant association between age group and CagA-positive infection. The lowest infection prevalence was found among patients in the 70-79 and 80-89 years age groups (2.78%) (Figure-2). Based on the study conducted by Lamichhane et al. [26], incidence of *H. pylori* decreases at ages >60 years old. The results indicated no significant association between age and *H. pylori* infection. However, another study conducted in a different population showed a higher proportion of *H. pylori* infection among those aged <29 years and those within the 50-59 years of age group [27]. These results are also supported by other research that found a similar tendency of the rates of *H. pylori* infection among individuals aged >40 years [28]. In general, the prevalence of *H. pylori* infection throughout different time periods is 74.7%, 53.0%, and 35.1% in the 1970s, 1990s, and 2010s, respectively [29].

Here, the prevalence of CagA-positive *H. pylori* infection in Denpasar, Bali was higher when compared to other areas at 41.67% (15 patients). The relationship between demographic background and CagA-positive infection was statistically not significant with p=0.769. Thus, these results indicate that demographic characteristics do not influence the occurrence of *H. pylori* infection (Figure-3).

In this study, we found gastritis to be the most commonly detected gastric pathology (94.4%). The association between endoscopic results and CagA-positive infection was statistically insignificant with p=0.663, thus indicating that the theory of endoscopic alteration as being a sensitive indicator of *H. pylori* infection remains unelucidated (Figure-4). Another study also implicated gastritis (69.0%) as the most common gastric pathology detected during *H. pylori* infection [30]. Correlation between endoscopic alteration and *H. pylori* infection was statistically significant with p<0.01, indicating that endoscopic alteration could be used as a sensitive indicator for predicting *H. pylori* infection [31].

A majority of the study participants exhibited mild gastric inflammation. The relationship between the degree of inflammation and CagA-positive infection was not statistically significant (p=0.769) (Figure-5). A previous study described 35 (67.31%) patients had acute inflammation, which was comprised 29 (83%) mild, 5 (14%) moderate, and 1 (3%) severe inflammation. Meanwhile, 51 (98.08%) patients were found to have chronic inflammation, of which 2 (4%) exhibited mild, 14 (27%) moderate, and 35 (69%) severe inflammation. *H. pylori* infection was detected in 30 patients, with 3 (10%) presenting with mild, 6 (20%) with moderate, and 21 (70%) with severe disease [32].

### Variation of CagA protein

According to the phylogenetic analyses and the 12 CagA sequences obtained herein, it can be concluded that the *H. pylori* strain of Bali can be grouped within the East Asian genotypes and is identical to Lombok strain (Figure-6). In addition, these Bali isolates are phylogenetically more closely related to Southeast Asian strains of the bacteria, particularly the Filipino strain. From the phylogenetic tree, the cluster representing the *H. pylori* Bali strain is shown far from the East Asian cluster.

**Figure-7:**
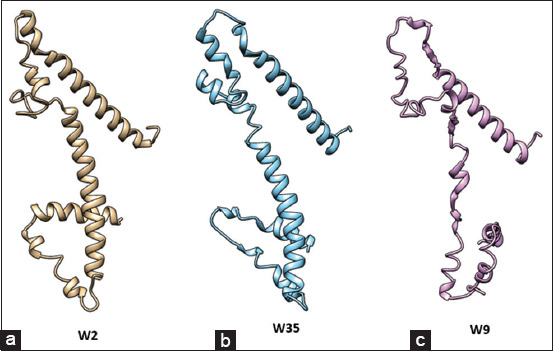
Prediction of protein structure of the C-terminal cytotoxic associated antigen A fragment of *Helicobacter pylori* Bali isolates. (a) W2 fragment with the EPIYA-ABD (East Asian) motif in patient with gastric cancer; (b) W35 fragment with EPIYA-ABD (East Asian) motif in gastritis patients; and (c) W9 fragment with EPIYA ABC (Western) motif.

The *H. pylori* strain from Bali also contained an EPIYA amino acid coding motif within the *CagA* gene that underwent substitution of its amino acids from A to T, hence, transforming it into an EPIYT motif (Figure-7). This EPIYT motif is suspected to be more virulent and increases the risk of developing cancer [33,34]. CagA-positive *H. pylori* infection induces alteration of inflammation levels of gastric mucosa, which in turn eventually can cause gastric cancer, superficial gastritis, atrophic gastritis, intestinal metaplasia, dysplasia, and carcinoma [35].

This study employed PCR using DNA from corpus and antral tissues to detect CagA protein. As mentioned, the prevalence of CagA-positive results found here was 33.3%. A previous study found similar results with 32% of gastric cancer patients being CagA-positive [36]. A Japanese group that also used PCR as their confirmatory modality found 31.6% CagA-positive cases from 57 gastric cancer tissues [37]. Meanwhile, a study conducted in South Korea also showed a high prevalence of CagA-positive cases [38]. Overall, it was found that 22.4% (n=37/165) patients were infected with CagA-positive *H. pylori* strains and 77.6% (n=128/165) patients were infected with a CagA-negative strain [29].

Two primary forms of CagA protein have been identified: East Asian and Western. In East Asian countries – such as Japan, South Korea, and China – most *H. pylori* infections were of the East Asian CagA strain. Level of inflammation, gastritis activity, and atrophy was significantly higher among patients infected with the CagA-positive East Asian strain as compared to those infected with CagA-negative East Asian or CagA-positive Western strains [39].

As the most relevant virulence factor of *H. pylori*, CagA interferes with cellular function through physical interaction and intracellular signal deregulation both through dependent tyrosine phosphorylation and an independent mechanism after its delivery to gastric epithelial cells [40]. After being translocated into the host cytoplasm, CagA binds the surface of the cell membrane and undergoes tyrosine phosphorylation. The phosphorylated and unphosphorylated forms of CagA interact with a host protein to activate downstream signaling pathways, such as induction of ornithine decarboxylase upregulation through the Src/MEK/ERK/c-Myc pathway [41] and trafficking of REG3γ within gastric epithelial cells by activation of the interleukin (IL)-11/gp130/STAT3 pathway [42]. Non-phosphorylated CagA protein can activate hepatocyte growth factor/c-Met receptor scatter factor and adaptor Grb2 protein, induce phosphorylation of gamma phospholipase C and impair formation of e-cadherin/b-catenin formation, and affect inhibition of polarity-regulating kinase partitioning-defective 1b/microtubules kinase-2 to interfere with the atypical signaling pathway of protein kinase C.

**Figure-8:**
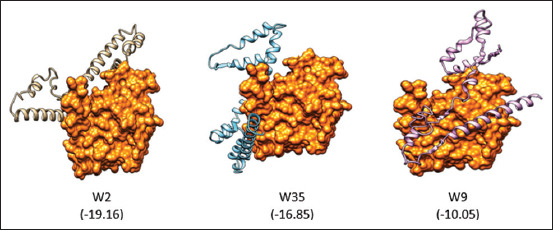
*In silico* analysis for the interaction between C-terminus of the cytotoxic associated antigen A protein fragment and SHP2. The numbers in the bracket represent free energy binding. The more negative the free energy binding, the stronger the binding between a ligand and its receptor.

Experiments with transgenic zebrafish revealed the wild-type and phosphorylation-resistant forms of CagA that showed significantly increased intestinal epithelial proliferation and upregulation of several target genes: Wnt cyclinD1, axin2, and zebrafish c-myc ortholog myca [43]. In addition, CagA was also found to induce higher production of IL-8, nuclear factor κB (NF-κB), activator protein-1 and fatty acid dislocase (FAT) activities [44] and increased the activity of transforming growth factor beta-activated kinase 1 (TAK1) and KIA-mediated induction of TAK1-NF-κB that was associated with KIA and also connected to TPA1, which is eventually utilized by the CagA protein of *H. pylori* to induce an inflammatory response [45]. This might also inhibit miR-370 expression, which can lead to overexpression of FoxM1 and subsequently increase intestinal cell proliferation [46]. These findings implicated some important roles of CagA in gastric carcinogenesis. *In silico* analysis showed that CagA protein with an EPIYA-ABD motif exhibits stronger affinity toward SHP2 (Figure-8).

## Conclusion

The *H. pylori* Bali strain consists of the East Asia genotype and possesses identical characteristics of the Lombok strain. In addition, Bali isolates are phylogenetically more closely related to Southeast Asian strains of the bacteria, in particular the Filipino strain. From the phylogenetic tree, it is shown that the *H. pylori* strain cluster of Bali origin is distant from the East Asian cluster.

## Authors’ Contributions

HS: Designed the research, lab work, analyze the data, and writing the manuscript; DNW: Designed the research, sample collection, laboratory work, analyze the data, and writing the manuscript; ZM: Sample collection, laboratory work, analyzed the data, and writing the manuscript; KKA: Sample collection, laboratory work, analyze the data, and writing the manuscript. All authors read and approved the final manuscript.
